# An empirical study of older adults’ willingness and enthusiasm for informal learning

**DOI:** 10.3389/fpsyg.2026.1782394

**Published:** 2026-08-06

**Authors:** Guoyi Lai, Mengting Han

**Affiliations:** 1 Department of General Education, Sichuan Post and Telecommunication College, Chengdu, China; 2 International Education College, Chengdu Normal University, Chengdu, China

**Keywords:** older adults, informal learning, willingness to participate, enthusiasm for participation, empirical research

## Abstract

**Introduction:**

As global populations age, promoting informal learning among older adults is crucial for their well-being and social participation. However, research on older adults’informal learning remains limited, especially in non-Western contexts. This study investigates the current status, willingness, and enthusiasm of Chinese older adults for informal learning, as well as the influencing factors from individual, cognitive, and environmental perspectives.

**Methods:**

Using cross-sectional data from the 2018 China General Social Survey (CGSS), this study analyzed 4,688 respondents aged 60 years and older. Informal learning was measured by reading behavior (books/newspapers/magazines) in spare time. Binary logistic regression and multinomial ordered logistic regression were employed to examine factors associated with participation willingness (binary) and participation enthusiasm (ordinal frequency), based on Bandura’s social cognitive theory.

**Results:**

Only 18.47% of older adults participated in literacy-based informal learning. Men, individuals with a spouse, higher education, better health, and higher life satisfaction showed greater willingness. Mandarin proficiency and social interaction frequency significantly promoted both willingness and enthusiasm. Notably, Mandarin proficiency had the strongest positive effect on participation enthusiasm (odds ratios ranging from 1.33 to 2.22 across frequency levels). Economic status and social trust showed no significant effects.

**Discussion:**

Chinese older adults exhibit low willingness and enthusiasm for informal learning. Cognitive factors (especially language ability) and social interaction are more influential than economic resources. The findings suggest that policies should enhance Mandarin training and community-based social networks, with tailored strategies for married vs. unmarried older adults. Future research should adopt longitudinal designs and broader measures of informal learning (digital, social, and experiential).

## 1 Introduction

From a practical perspective, older adults’ participation in learning contributes to improving their cognitive abilities and health literacy, thereby increasing human capital. This, in turn, contributes valuable human resources to the labor market and promotes the development of industries associated with older adult education. Besides, participating in learning meets older adults’ growing desire to enrich their later years and fulfill their need to realize life value (Jenkins and Mostafa, 2013).

Globally, in the field of older adult education research, the government-led model has become mainstream. As a result, many countries focus their research primarily on formal educational models such as community-based older adult education and universities of the third age. For example, Makino (2013) examines grassroots communities and lifelong learning in Japan, aiming to connect learning activities with new communities and to make “learning” a key task for the government in community self-governance. Tseng and Wu (2018) argue that geographic location is a decisive factor influencing older adults’ participation in social activities. Using the Gini coefficient, they discuss the disparities and inequalities in older adults’ access to community-based learning resources caused by geographic location. They suggest that geographic location, the number of older adults with learning needs, and the supply capacity of community learning centers should be comprehensively considered in future community-based older adult learning programs. Cook (2012) studied intergenerational cooperation in senior colleges in the United States and Hong Kong. Additionally, some scholars have explored older adult education through higher education and senior care facilities. For instance, Hietanen et al. (2012) investigated the development and current state of higher education in gerontology in Nordic countries, while Boscart et al. (2020) studied nursing education models. However, from a practical perspective, participating in formal education is only a choice for some older adults. Research (Villar and Celdrán, 2013; Hansen et al., 2019) indicates that in Western countries, older adults in the United States participate in learning activities most frequently, followed by those in Nordic countries, with Western European countries showing the least participation. The reason for this variation lies in the fact that formal education requires fixed locations, designated teachers, and well-designed curricula (Livingstone, 2001). With the rapid evolution of digitalization, knowledge expands exponentially, increasing the need for continuous learning to avoid social obsolescence. Given that most knowledge is currently acquired through informal learning, this learning modality has grown increasingly essential. Consequently, research on informal learning has emerged as a frontier area of study.

Informal learning is clearly more meaningful for enhancing older adults’ later life, given its far higher prevalence compared to other learning forms (Villar and Celdrán, 2013). As Brockett and Hiemstra (1991) pointed out: “Education, and especially informal learning, has been positively related to higher life satisfaction in older adults.” However, globally, there is limited specialized research on informal learning among older adults, by both Chinese and foreign scholars. China has the largest older adult population. Studying the informal learning status of older adults in China would greatly enrich research in this field and provide valuable insights for the development of older adult education worldwide.

In summary, this study uses cross-sectional data from the 2018 China General Social Survey (CGSS) and employs cross-tabulation analysis and multiple regression analysis. This study primarily addresses the following two questions:

RQ1. What is the current status, willingness, and the influencing factors of informal learning among older adults?

RQ2. What is the enthusiasm of older adults for informal learning and the influencing factors?

The contributions of this paper 2-fold:

- It enriches research in the field of older adult education and fills the gap created by the lack of Chinese samples, providing scholars worldwide with an intuitive understanding of the status of Chinese older adults’ participation in informal learning.
- It offers policy support for the development of older adult education in emerging economies and provides a valuable Chinese model for the global advancement of lifelong learning.

The structure of the paper is as follows: First, a review of the relevant research in the field is presented. Then, the theoretical framework, survey data, research variables, and methodology are introduced. Based on these, the research findings are discussed, followed by implications.

## 2 Literature review and conceptual distinction

### 2.1 Literature review

Given the limited research specifically focused on informal learning for older adults, we expand the scope of our literature review to the entire field of older adult education, including formal, non-formal, and informal education. According to La Belle (1982), formal learning refers to structured learning that takes place within an organized, institutional setting, such as schools, universities, and vocational training centers. It is typically led by trained educators and follows a specific curriculum, often leading to a recognized qualification or certification. Non-formal learning is intentional and organized learning that takes place outside of formal educational institutions, and does not usually result in formal certification or qualification. Informal learning occurs naturally and spontaneously in daily contexts such as interacting with family or friends, reading books or newspapers, listening to the radio or watching TV, visiting museums, or attending lectures and conferences. It is not organized or planned.

Through a literature review, we identify three key aspects for examining the factors influencing informal learning in older adults: The first focuses on individual characteristics; the second examines the environmental factors in which older adults are situated; and the third analyzes the availability of educational resources.

First, from the perspective of individual characteristics of older adults, research suggests that the willingness to participate in learning is influenced by demographic factors such as gender, race, and educational level. Kim and Merriam (2004) conducted a study on 189 students aged over 50 from a retirement institute in the southeastern United States, using a survey to examine participants’ demographic characteristics, social interactions, family reunions, and cognitive interests. By comparing the scores of various survey items and analyzing correlations between different dimensions, the study concluded that highly educated middle-class white individuals were the primary participants in educational activities. Hansen et al. (2019) reached similar conclusions in their study. Punksungka et al. (2021) further found that highly educated, middle-class white women exhibited a stronger willingness to participate in learning activities. Meanwhile, Downing and Copeland (1980) noted that the participation willingness of Black older adults is relatively low, as reflected in their insufficient utilization of community educational resources.

Among demographic characteristics, educational level and socioeconomic status are two crucial factors that cannot be overlooked. Roman (2004) found in a study on an adult literacy program in the United States that the detrimental impact of illiteracy intensifies over the course of life, leading to considerable disadvantages for older adults. Bynum and Seaman (1993) discovered in their study of 452 older adults participating in Learning-in-Retirement institutes that these individuals generally had higher economic status and extensive educational backgrounds. Their participation was driven by factors such as self-actualization, recognition of cognitive gaps, intellectual curiosity, and the desire for social interaction.

Recent cohort studies have further refined our understanding of educational stratification in older adults’ learning. As Cruce and Hillman (2012) initially documented, the demand for higher education among older adults was already rising. Cruce and Hillman (2023), in a longitudinal analysis of U.S. higher education enrollment, found that the educational gradient in older adults’ learning has steepened over the past decade, with college-educated older adults increasingly likely to pursue continuous learning opportunities while those with high school or less education show declining participation rates. This trend suggests that informal learning may be becoming more unequally distributed alongside formal education expansion, a pattern that may be particularly pronounced in rapidly developing economies like China where educational expansion occurred unevenly across cohorts.

Additionally, older adults often prefer to learn in groups. Simson et al. (2002) pointed out that there are five areas that should be considered: (1) the background of peer educators; (2) the characteristics of their study groups; (3) the motivations and rewards of peer educators; (4) the training that peer educators acquire; and (5) the teaching-learning experience of peer educators. Indeed, the growing homogeneity among participants in older adult education has gradually garnered the attention of researchers.

The homogeneity of learning groups has gained renewed attention in recent research. Talmage et al. (2022), analyzing ten years of enrollment data from Osher Lifelong Learning Institutes, documented growing educational and socioeconomic homogeneity among participants, raising concerns about the “exclusionary potential” of even well-intentioned community-based learning programs. This finding is particularly relevant for understanding informal learning, where self-selection into social networks may reproduce rather than bridge social inequalities.

Furthermore, older adults’ participation in informal learning is also driven by individuals’ intrinsic interests. Tam (2016) found in a research with a group of Chinese older adults in Hong Kong that a strong interest in the content of learning programs as well as the desire to enrich themselves and their lives are important factors influencing older adults’ participation in learning. Helterbran (2017) conducted semi-structured interviews with older adults returning to university to earn a bachelor’s degree. After coding and analyzing the interview results, she concluded that the motivation for older adults’ participation motivation lies in the pursuit of enjoyment and personal enrichment. Therefore, older adults’ interest in learning content and the desire to enhance life satisfaction have emerged as shared driving forces behind their engagement in educational activities.

Second, from the perspective of the environment in which older adults live, macro-level institutions, technological conditions, cultural environments and personal resource availability all influence their willingness to learn. Boeren and Holford (2016) found that some factors, such as national macro-institutions and welfare systems have a more significant impact on older adults’ participation in learning.

From the perspective of the technological environment, Lai (2018) argues that older adults’ decisions to engage in learning behaviors are influenced by the social environment and accessibility conditions, particularly the perceived usefulness of mobile devices for learning. The digital transformation of informal learning has accelerated dramatically, a trend particularly highlighted by the COVID-19 pandemic. Lü et al. (2023), in a mixed-methods study of Chinese older adults during the pandemic, identified “digital learning resilience” as a key factor distinguishing those who maintained learning engagement from those who disengaged. However, they also documented a “double digital divide”—not merely access to devices, but the capacity to transform digital access into meaningful learning experiences. This finding underscores the limitation of our 2018 data in capturing the emerging digital informal learning ecosystem. Jenkins and Mostafa (2022), updating their earlier longitudinal analysis with additional waves of English data, confirmed that technology use for learning has become the fastest-growing predictor of cognitive engagement among older adults, surpassing traditional reading-based activities in predictive power for some age groups. This shift suggests that studies relying on pre-pandemic data may underestimate current informal learning prevalence and misidentify its key predictors.

From the perspective of the cultural environment, Findsen (2016) asserts that the two distinct cultural frameworks of European and indigenous peoples in New Zealand have infiltrated the policy practices of older adult education, with their learning participation being widely influenced by the socio-cultural context. Comparative research has increasingly emphasized cultural specificity in older adults’ learning. Findsen and Formosa (2023), in their comprehensive review of “learning cultures” in aging across 15 countries, argued that Confucian heritage cultures (including China, Korea, Japan, and Vietnam) exhibit distinct patterns characterized by stronger family-centered learning, greater emphasis on intergenerational knowledge transmission, and different valuations of “productive” versus “leisure” learning. These cultural specificities challenge the universal applicability of Western-derived theoretical frameworks and highlight the need for context-sensitive research instruments.

From the perspective of older adults’ resource environment, the primary influencing factors are family resources, as well as the social and human capital they possess. Cincinnato et al. (2016) assessed the impact of social background on adult education participation based on the theory of cultural capital. Using data from the Adult Skills Survey of 23 countries, a multiple regression model was established. They found that “educational practices are affected by one’s social background, and more specifically, by the cultural resources passed down within the family context.” Knipprath and De Rick (2015) used longitudinal data on educational participation across different age groups in Netherlands and applied multiple regression analysis to examine the impact of social capital on educational participation. The results showed that “Human capital, labor market position, and other individual characteristics are more important predictors of lifelong learning than social capital, but social capital can still be beneficial for those who do not have a higher educational degree.” The mechanisms through which social capital facilitates learning have been clarified by recent longitudinal research. Knipprath and De Rick (2022), extending their earlier Dutch study with 10-year follow-up data, found that network diversity (range of social roles and contexts) predicts learning participation more strongly than network size or network density. This suggests that informal learning benefits not merely from “being connected” but from exposure to diverse knowledge sources and learning opportunities across multiple social domains—a nuance not fully captured by our single-item social interaction measure. Findsen (2016) used a case study method to demonstrate the significant impact of the two distinct social cultures of European and indigenous populations in New Zealand on older adults’ participation in education. According to these researches, individual’s resource environment factors such as family background, social capital, and cultural context are not unique proxy variables. The impact of society on older adults’ participation in education requires further research.

Thirdly, research from the perspective of the supply of learning resources for older adults tends to focus on specific educational institutions or programs. Studies have indicated that the mission and vision of older adult education institutions, as well as the design of learning content and the organization of learning activities, are important factors that influence older adult participation in education. For example, Talmage et al. (2015) noted that “lifelong learners show more interest in classes related to global issues, religion/philosophy, and social issues that focus on specific groups and individuals.” Ollis et al. (2018) used a case study approach to investigate the learning experiences of older adults returning to education, outlining the benefits of health, wellbeing, knowledge, and skill development through formal, informal, and incidental learning in neighborhood houses. Additionally, Rebecca et al. (2016) and Hachem and Formosa (2023), in a study of a university for older adults in the northeast of the UK, found that institutions’ insufficient understanding of their goals and roles, as well as inconvenient physical facilities can hinder low-income groups from engaging in education. Formosa (2014) and Hachem et al. (2017), in their studies of other older adult universities, also found that the institution’s mission, inclusive membership systems, and empowerment of older adult directly influence their participation in education.

The COVID-19 pandemic forced rapid adaptation in older adult education provision, with important implications for informal learning. Boscart et al. (2022) and Hachem et al. (2023), Hachem (2023a) documented how nursing homes and community centers in Lebanon and Canada, respectively, transformed informal learning activities through hybrid delivery models combining in-person and digital engagement. These adaptations revealed both the resilience of informal learning (its capacity to persist despite institutional disruption) and its fragility (dependence on sustained social infrastructure). The post-pandemic “new normal” for older adult learning remains inadequately understood, particularly in non-Western contexts.

Critical gerontology perspectives have increasingly questioned the emancipatory narrative of older adult education. Formosa and Hachem (2024), synthesizing two decades of critical educational gerontology research, argued that “universities of the third age” and similar institutions often reproduce rather than challenge existing social inequalities, serving primarily the “young-old, healthy, educated, and affluent.” They call for greater attention to “learning in the margins”—informal, often invisible learning practices of disadvantaged older adults. Our focus on nationally representative data, including non-participants, responds partially to this call, though our literacy-based measure still privileges visible, individual learning forms.

In summary, although previous studies have explored the issue from various perspectives, limitations remain. According to social cognitive theory, human behavior, individual cognitive factors, and environmental factors are dynamically interconnected and mutually determining (Bandura, 1986). In this interactive system, specific behaviors, such as individual actions or choices, are the result of the interplay between external environments and internal cognition. External environments encompass both physical resources and interactions with others, while internal cognition reflects an individual’s attitudes, knowledge, or beliefs. Therefore, the behavior of older adults participating in learning must be examined within this interactive system. Previous researches often approached the topic from a single perspective, isolating cognitive and environmental factors, which makes it challenging to draw more robust conclusions.

Recent theoretical developments have sought to update social cognitive theory for the digital age. Lai and Smith (2023) proposed an extended social cognitive model of older adult learning incorporating digital self-efficacy, technology anxiety, and online social support as key constructs. Their model, tested with Taiwanese older adults, explained substantially more variance in learning engagement than traditional SCT models, suggesting that technological capability beliefs have become as important as traditional self-efficacy domains. This theoretical extension is particularly relevant for interpreting our Mandarin proficiency findings, which may partially proxy for broader digital-literacy capabilities not directly measured in our 2018 data.

Additionally, from the perspective of research samples, previous studies have often selected specific older adult education institutions or educational programs in particular regions, which has led to potential biases in the research. Sampling from individuals participating in senior universities or specific educational programs overlooks the reality of insufficient educational resources and the unequal distribution of educational opportunities, which is a significant factor influencing older adults’ engagement in lifelong learning. Chen and Wang (2024), in a national survey of Chinese universities for the aged, found that current participants remain predominantly urban, educated, and healthy—confirming the selective nature of formal older adult education and underscoring the need to study informal learning among those excluded from these institutions. Therefore, a broader, large-scale sample would be beneficial for a deeper exploration of the factors affecting older adults’ participation in informal learning.

Based on Bandura’s (1986) social cognitive theory, this study selects relevant variables related to individual characteristics, cognitive-behavioral factors, and environmental factors. The study employs cross-tabulation analysis to descriptively examine older adults’ participation in informal learning, comparing demographic differences between participants and non-participants. Additionally, multiple regression analysis is used to examine the factors influencing older adults’ engagement in informal learning.

#### 2.1.1 Conceptual boundaries and debates in informal learning research

Informal learning is distinguished from formal and non-formal education by three core features: it operates without externally imposed curricular criteria, does not lead to formal certification, and is embedded in daily life contexts (La Belle, 1982; Livingstone, 2001). However, prior research exhibits two problematic tendencies. First, conceptual overreach—treating all non-institutional learning activities as informal learning, which blurs theoretical boundaries. Second, theoretical misalignment—applying theoretical frameworks developed for formal education to explain informal learning phenomena, thereby neglecting its unique mechanisms (spontaneity, self-regulation, social embeddedness).

This study adopts a narrow operational stance: informal learning is defined as self-initiated, non-curricular activities aimed at acquiring knowledge, skills, or understanding. Under this definition, reading behavior—as a prototypical self-directed, literacy-based cognitive activity—serves as an archetypal form of informal learning. However, we acknowledge that this operationalization excludes other valid forms such as oral/aural learning, digital learning, and experiential learning. This limitation is discussed in detail in the methodology section.

Furthermore, prior research exhibits systematic measurement bias: most studies sample from formal educational institutions (e.g., universities of the third age), resulting in samples biased toward already-engaged participants and failing to identify structural barriers to informal learning (OECD, 2022). By using nationally representative survey data that includes participants and non-participants, our study partially overcomes this bias.

### 2.2 Identified research gaps

Recent reviews confirm persistent gaps. OECD (2022), in its comprehensive report on “Aging and Skill Development,” explicitly noted the lack of large-scale representative data on informal learning among older adults in middle-income countries, identifying this as a critical barrier to evidence-based policy. European Commission (2021), evaluating progress toward the European Agenda for Adult Learning, similarly highlighted that “invisible learning”—self-directed, non-institutional learning—remains systematically undermeasured in official statistics, leading to policy neglect of the most prevalent form of older adult learning. Our study directly addresses these identified measurement and evidence gaps.

Gap 1: Geographic bias in sampling. Existing studies predominantly sample from formal educational institutions (e.g., Villar and Celdrán, 2013; Hansen et al., 2019) or specific community programs (e.g., Tseng and Wu, 2018), systematically excluding the majority of older adults who do not access these resources. This creates a survivorship bias toward the already-engaged, obscuring understanding of barriers faced by non-participants.
Gap 2: Conceptual conflation of learning modalities. Prior research often treats formal, non-formal, and informal learning as a unified category (e.g., “lifelong learning participation”), failing to recognize that informal learning operates through distinct mechanisms—spontaneous, self-directed, and embedded in daily life (Livingstone, 2001; La Belle, 1982). This conflation limits theoretical precision.
Gap 3: Underrepresentation of Eastern, particularly Chinese, contexts. While China possesses the world’s largest older adult population, rigorous empirical studies on Chinese older adults’ informal learning remain scarce in international literature. Existing comparative studies (e.g., Villar and Celdrán, 2013) exclude China, and domestic studies in China often lack methodological rigor or theoretical framing. Zhang and Li (2022), in a qualitative study of rural Chinese older adults, provided rich insights into community-based learning practices but lacked national representativeness and theoretical framing—limitations our study addresses through large-scale survey data and explicit social cognitive theory grounding. This creates a knowledge void regarding how cultural, linguistic, and institutional specificities shape informal learning in non-Western contexts.

How this study addresses these gaps: By utilizing nationally representative survey data that includes both participants and non-participants, we overcome institutional sampling bias (Gap 1). By explicitly focusing on informal learning and distinguishing it from formal modalities, we enhance conceptual precision (Gap 2). By providing theoretically grounded, methodologically rigorous evidence from China, we fill the geographic knowledge void (Gap 3).

### 2.3 Conceptual distinction between willingness and enthusiasm

In the context of older adults’ informal learning, we theoretically distinguish between participation willingness and participation enthusiasm based on the behavioral engagement literature (Gagné and Deci, 2005; Ryan and Deci, 2017; Hachem, 2023b).

Participation willingness refers to the binary behavioral intention to engage in informal learning activities—whether an individual chooses to participate at all. It captures the extensive margin of participation. This concept aligns with the ‘decision to participate’ stage in the engagement process, reflecting an individual’s motivational readiness and absence of barriers to entry.

Participation enthusiasm refers to the intensive behavioral engagement—the frequency, intensity, and sustained nature of participation among those who have already decided to participate. It captures the intensive margin of participation. This concept reflects the “quality of engagement” and intrinsic motivation once the participation decision has been made.

Theoretical rationale for this distinction: Drawing from self-determination theory (Deci and Ryan, 2000), willingness is primarily associated with controlled motivation (external and introjected regulations)—driven by perceived utility, social pressure, or lack of barriers. Enthusiasm, in contrast, is associated with autonomous motivation (identified and intrinsic regulations)—driven by inherent enjoyment, interest, and personal value of learning. This conceptual distinction allows us to test whether different factors predict the decision to participate versus the intensity of engagement.

### 2.4 Critical assessment of operationalization

We acknowledge that our measurement represents a behavioral proxy rather than direct attitudinal assessment. Willingness is operationalized as the dichotomous response to participation (yes/no), which captures behavioral manifestation of willingness but may miss the attitudinal formation process. Enthusiasm is operationalized as ordinal frequency categories, which approximates intensity but conflates frequency with emotional engagement.

Ideally, both constructs would be measured using multi-item scales assessing attitudinal, emotional, and behavioral components [e.g., the Adult Engagement Scale developed by Khan et al., 2024]. However, within the constraints of secondary data analysis using CGSS, our operationalization represents the best available behavioral approximation. We interpret “willingness” as “manifest behavioral willingness” and “enthusiasm” as “behavioral intensity,” with appropriate caveats regarding the attitudinal-emotional components not directly captured.

### 2.5 Narrow operationalization of informal learning: a proxy indicator approach

We acknowledge that our measurement of informal learning is necessarily narrow due to data constraints. The 2018 CGSS wave contains only a single item (a36) assessing reading behavior: “Did you often read books/newspapers/magazines in your spare time in the past year?” with response categories from “never” to “very often.”

Theoretical justification for using reading as a proxy:

Reading represents the archetypal form of self-directed, literacy-based informal learning (Livingstone, 2001; La Belle, 1982). It is:

**Table:** 

Excluded learning form	Definition	Example activities	Population affected
Social learning	Knowledge acquisition through interpersonal interaction	Family discussions, peer counseling, intergenerational skill sharing	All older adults, especially those in multigenerational households
Digital learning	Self-directed learning via internet/mobile devices	Health information seeking, online courses, short-video learning	Urban, younger-old, higher-educated cohorts
Experiential learning	Learning through direct experience and reflection	Travel learning, volunteering, hobbyist practice	Active, healthy older adults
Embodied learning	Skill development integrated with physical activity	Tai chi philosophy, traditional medicine practice, craft-making	Physically active older adults
Community-based learning	Participation in organized but non-curricular activities	Community lectures, cultural performances, neighborhood study groups	Urban residents with community access

- Self-initiated: Requires voluntary engagement without external curriculum.
- Self-regulated: Pace and depth determined by learner.
- Contextually embedded: Occurs in daily life spaces (home, community).
- Knowledge-seeking: Explicitly oriented toward information/understanding acquisition.

However, we recognize that this operationalization systematically excludes other valid forms of informal learning identified in the literature (Villar and Celdrán, 2013; Biesta, 2011a):

Consequence of exclusion: Our measure likely underestimates true informal learning prevalence, particularly among:

- Rural populations with oral/aural learning traditions.
- Low-literacy individuals relying on radio/TV/video.
- Digitally-engaged cohorts using mobile learning platforms.
- Physically-active individuals learning through practice.

## 3 Methodology

### 3.1 Analytical strategy

This study employed a quantitative research design, utilizing cross-sectional survey data from the 2018 China General Social Survey (CGSS). The analytical strategy proceeded in two stages. First, we employed descriptive statistical analysis (cross-tabulation with χ^2^ tests) to examine the current status of older adults’ informal learning participation and identify significant demographic differences between participants and non-participants. Second, we used inferential statistical methods—binary logistic regression and multinomial ordered logistic regression—to examine the directional effects and relative magnitude of influence exerted by Individual Factors (IF), Environmental Factors (EF), and Cognitive Factors (CF) on participation willingness and participation enthusiasm, respectively.

Firstly, we analyzed the current status of older adults’ informal learning, in order to identify older adults’ informal learning willingness and whether different demographic characteristics significantly impact their willingness to participate and the level of their active participation, by using Cross-Tabulation Analysis, a descriptive statistical method. We adopt a χ^2^ test with a significant level of 0.05 to confirm the correlation between the variables and the independent variable. Then, we answered the first research question.

Secondly, we examined the direction and extent of the influence of Individual Factors (IF), Environmental Factors (EF), and Cognitive Factors (CF) on their participation willingness and participation enthusiasm, by using Multiple Regression Analysis. The Binary dependent variables are Participation and Non-Participation.

In the regression analysis, a Binary Logistic Regression Model was used to estimate the likelihood of participation in informal learning based on various predictors such as individual factors, environmental abilities, and cognitive factors. In the estimation process, we employed hierarchical model specification grounded in Bandura’s (1986) social cognitive theory rather than data-driven stepwise selection. The modeling proceeded in three theoretically justified stages:

Model 1 (baseline): Individual demographic factors (age, gender, education, marital status, urban/rural residence, self-rated health) were entered first to establish the foundational effects of personal characteristics.
Model 2 (cognitive extension): Cognitive factors (life satisfaction, Mandarin proficiency, depression status) were added to examine whether psychological and linguistic capabilities explained additional variance beyond demographics.
Model 3 (full model): Environmental factors (income level, social trust, social interaction frequency) were incorporated last to test the incremental contribution of contextual variables after accounting for individual and cognitive factors.

This hierarchical approach aligns with the theoretical precedence of personal factors → cognitive processing → environmental influence in social cognitive theory, ensuring model specification was theory-driven rather than data-driven.

Binary logistic regression model:


$$\mathrm{logit}\,\left(p\right)=L\,n\,\left(\frac{p}{1-p}\right)=\alpha +\beta_{1}\,I\,F+\beta_{2}\,E\,F+\beta_{3}\,C\,F+\varepsilon$$

Note: β_1_ β_2_ and β_3_ means the estimated parameters; α means constant; ε means Random Error.

In order to mitigate the loss of sample information caused by binary variables, this research employs a multinomial ordered logistic model to estimate the factors influencing the “the participation willingness of informal learning.” The Ordered variables are divided into five levels: Never/Rarely/Sometimes/Often/Very often. We used the maximum likelihood method in SPSS 20.0 to obtain estimated coefficients and perform significance tests of the regression coefficients. Then, we answered the second research question.

Multinomial ordered logistic model:


$$\text{logit}\,\left(p_{y\leq k}\right)=L\,n\,\frac{p_{y\leq k}}{1-p_{y\leq k}}=\alpha_{k}+\overset{m}{\underset{i=1}{\sum }}\beta_{i}\,X_{i},k=1,2,\ldots .n-1$$

Note: Xi represents the i-th indicator variable; n denotes the number of classification levels.

#### 3.1.1 Rationale for social cognitive theory and variable mapping

Social cognitive theory (Bandura, 1986) posits that behavior, personal cognitive factors, and environmental factors constitute a dynamic triadic reciprocal determinism. This study adopts this theoretical framework for two reasons.

First, informal learning lacks external curricular structures; its occurrence depends heavily on individuals’ self-efficacy and cognitive capabilities (e.g., language processing, information acquisition). Second, informal learning is embedded in daily environments, where social interaction and resource accessibility directly shape learning opportunities. Social cognitive theory provides an integrated framework that accommodates all three domains.

Accordingly, we map theoretical constructs to observable variables as follows:

**Table:** 

Theoretical construct	Operationalized variable	Measurement content
Personal factors	Gender, age, education, marital status, urban/rural, health	Ascribed characteristics
Cognitive factors	Mandarin proficiency, life satisfaction, depression	Language processing, subjective well-being, emotional resources
Environmental factors	Income level, social trust, social interaction frequency	Economic resources, perceived environmental safety, social engagement

The hierarchical entry order of the three models (persona → cognitive → environmental) follows the theoretical logic: personal characteristics constitute foundational endowments, cognitive factors serve as mediating mechanisms, and environmental factors function as moderating conditions. This theory-driven specification contrasts with data-driven stepwise selection.

### 3.2 Model diagnostics

We conducted comprehensive diagnostic tests to ensure the validity of statistical inferences:

Multicollinearity assessment: Variance Inflation Factors (VIF) were calculated for all predictor variables. All VIF values were below 2.5 (range: 1.02–2.34), well below the conventional threshold of 10, indicating no serious multicollinearity concerns.
Proportional odds assumption: For the multinomial ordered logistic regression, we tested the parallel lines assumption using the Brant test. The overall test was non-significant (χ^2^ = 23.47, *p* = 0.214), supporting the validity of the proportional odds assumption. Variable-specific tests revealed minor violations for education level (*p* = 0.042); however, given the theoretical importance of education and its marginal violation, we retained it with appropriate interpretive caution.
Model fit indices: We reported Nagelkerke R^2^ for all models as an indicator of explained variance. Additionally, we calculated Akaike Information Criterion (AIC) and Bayesian Information Criterion (BIC) for model comparison, with lower values indicating better fit.
Goodness-of-fit: For binary logistic models, Hosmer-Lemeshow tests yielded non-significant results (*p* > 0.05), indicating adequate calibration. For ordered logistic models, we examined pseudo-R^2^ values and model likelihood ratio tests (all *p* < 0.001).

Table 1 summarizes the diagnostic statistics, including multicollinearity (VIF range 1.02–2.34), proportional odds assumption (Brant test χ^2^ = 23.47, *p* = 0.214), and model fit indices. More messages see Supplementary Appendix A.

**TABLE 1:** Summary of regression model diagnostic statistics.

Diagnostic item	Index/test	Result	Conclusion
Multicollinearity	VIF range	1.02–2.34	No severe multicollinearity
Proportional odds assumption	Brant test (overall)	χ^2^ = 23.47, *p* = 0.214	Assumption holds
Binary model fit	Hosmer-Lemeshow test	*p* > 0.05	Good model calibration
Ordinal model fit	Likelihood ratio test	*p* < 0.001	Model significant overall
Model comparison	AIC/BIC	Reported for each model	Model 3 is optimal

## 4 Data collection

This study utilizes data from the China General Social Survey (CGSS)^1^. Individuals aged 60 and above were selected as the participants, yielding a total of 4,688 samples.

Launched in 2003, the Chinese General Social Survey (CGSS) is the first national representative continuous survey project run by National Survey Research Center at Renmin University of China (NSRC) in mainland China. CGSS is aimed to systematically monitor the changing relationship between social structure and quality of life in both urban and rural China.

The Comparative Advantages of CGSS for Studying Older Adults’ Informal Learning over alternative data sources:

### 4.1 Specific strengths of CGSS 2018

**Table:** 

Data source	Key limitations	CGSS relative advantage
CHARLS (China Health and Retirement Longitudinal Study)	Focuses on health and retirement; lacks detailed learning behavior measures	CGSS includes comprehensive learning, social, and cultural participation items
CLHLS (Chinese Longitudinal Healthy Longevity Survey)	Sample concentrated in oldest-old (80+); cannot represent all older adults	CGSS covers full age range 60+ with representative age structure
Specialized older adult education surveys	Samples limited to educational institution participants; selection bias	CGSS household-based sampling includes non-participants
International surveys (e.g., SHARE, HRS)	Exclude China or Chinese samples lack national representativeness	CGSS provides nationally representative China-specific evidence

(A) Comprehensive coverage of theoretical constructs: Beyond the focal learning variable, CGSS includes rich measures of all three theoretical domains in Bandura’s (1986) framework—individual demographics, cognitive/psychological states (life satisfaction, depression), and environmental factors (social trust, interaction frequency, economic status)—enabling holistic testing of social cognitive theory.
(B) Linguistic and cultural specificity: The survey captures Mandarin proficiency as a key variable, which is theoretically crucial in China’s multilingual context but absent in Western datasets. This allows examination of language-as-barrier in informal learning, a significant contribution to understanding learning in linguistically diverse societies.
(C) Urban-rural stratification: CGSS’s explicit urban/rural sampling enables examination of China’s dualistic social structure, where educational resources and learning cultures differ dramatically. This is essential for policy relevance given China’s ongoing rural revitalization and aging-in-place initiatives.
(D) Temporal positioning: The 2018 wave captures a critical transition period—before the COVID-19 pandemic fundamentally altered learning modalities, yet during rapid digitalization expansion. This provides a baseline for understanding pre-pandemic informal learning patterns against which subsequent changes can be measured.

The latest sampling design made in 2018 used 2009 national population data as sampling frame. The design is Multi-Stage Stratified Design. There are three sampling stages: Primary sampling units (PSUs) is county-level units, there are 2762 PSUs in the sampling frame. Secondary sampling units (SSUs) are community-level units [villages (cun) and neighborhood committees (ju wei hui)]; in selected SSU, 25 households as Third-level sampling units (TSUs) are sampled with Probability Proportionate to Size Sampling (PPS) method; in each selected household, 18 and above adult will be sampled with Kish grid. There are 43 Municipalities directly under the central government, provincial capital cities, and vice provincial cities in China. Based on a comprehensive ranking of these cities by GDP, FDI, and education level, the top five are Beijing, Shanghai, Tianjin, Guangzhou, and Shenzhen. The 2018 design set these five cities as self-representative stratum. This stratum consists of 67 PSUs. The rest 2695 PSUs are comprehensively ranked with GDP per capital, urbanization rate, and population density. Then they are equally classified into 50 strata. Within each stratum, two PSUs will be selected with PPS method. In each selected PSU, four communities are sampled with PPS method. There are 80 communities in self-representative stratum and 400 communities in the rest 50 strata. The total sample size of 2018 design is 12,000 households and 2,000 are in self-representative stratum. More messages see Supplementary Appendix B.

## 5 Variables

### 5.1 Limitations of the dependent variable measurement

We explicitly characterize our measurement as a proxy indicator with three specific limitations:

#### 5.1.1 Why reading behavior as a proxy for informal learning?

Ideally, informal learning measurement should encompass multiple dimensions: literacy-based, digital, social, and experiential learning (Villar and Celdrán, 2013). However, under the constraints of secondary data analysis, this study relies on the only available relevant item in CGSS 2018 (a36: “Frequency of reading books/newspapers/magazines in spare time in the past year”). We justify using reading behavior as a proxy based on the following theoretical rationales:

First, self-directedness: Reading is voluntarily initiated by individuals without external curricula or instructors, aligning with the core definition of informal learning (Livingstone, 2001). Second, cognitive goal-orientation: Reading is typically undertaken for information acquisition, understanding, or knowledge accumulation, distinguishing it from pure entertainment. Third, contextual embeddedness: Reading occurs in daily life spaces such as homes and communities, without reliance on specialized educational facilities.

Furthermore, Wu and Jiang (2025) factor analysis of CGSS data demonstrated that the “learning and enrichment” factor (including reading books/newspapers/magazines, listening to music, physical exercise, and internet use) correlated with self-reported learning behavior at i = 0.53 (*p* < 0.001), providing empirical support for reading as a valid indicator of learning engagement.

However, we must explicitly acknowledge the measurement limitation: our findings pertain only to literacy-based informal learning and should not be generalized to digital, oral/aural, or experiential learning. Throughout the paper, we therefore use the qualifier “literacy-based informal learning” when interpreting results.

Limitation 1: Construct underrepresentation. The single-item reading measure captures only literacy-based, individual, indoor informal learning. It does not assess:

- Digital informal learning (e.g., smartphone-based health information seeking, WeChat group learning).
- Social informal learning (e.g., knowledge exchange in social gatherings, family storytelling).
- Experiential informal learning (e.g., learning through travel, volunteering, craft practice).

Implication: Our 18.47% participation rate should be interpreted as a lower-bound estimate of literacy-based informal learning, not the full scope of older adults’ informal learning engagement.

Limitation 2: Conflation of learning with entertainment. The survey item does not distinguish:

- Learning-oriented reading: Current affairs, health information, practical skills (e.g., cooking, gardening guides).
- Entertainment-oriented reading: Novels, entertainment news, leisure magazines.

Mitigation: We partially address this by noting that even entertainment reading may contribute to cognitive engagement and cultural participation, which are valued outcomes in gerontological education (Biesta, 2011a). However, we cannot isolate “pure learning” from “leisure consumption.”

Limitation 3: Literacy bias and digital exclusion. The measure systematically excludes:

- Illiterate or low-literacy older adults (27.54% of our sample reported no formal education).
- Non-readers who learn through oral/aural channels (radio, TV, conversation).
- Digital learners whose primary informal learning occurs via mobile devices.

Sensitivity check: We re-estimated all models excluding the illiterate group (*n* = 1,291). Results remained substantively unchanged (see Supplementary Appendix C), suggesting that within the literate population, the measure captures meaningful variation. However, this does not resolve exclusion of non-reading learning forms among the literate.

### 5.2 Dependent variables

According to the research of Livingstone (2001), informal learning is any activity involving the pursuit of understanding, knowledge or skill which occurs without the presence of externally imposed curricular criteria. It occurs naturally in the course of individuals’ production and daily life activities, facilitated by non-teaching social interactions. Informal learning is initiated, regulated, and self-directed by learners themselves, characterized by spontaneity, self-regulation, flexibility, social interaction, and contextual relevance.

The CGSS surveys the frequency of learning activities in spare time during the past year among older adults. Based on the definition of informal learning, we found this survey and its related questions, such as “Did you often read books/newspapers/magazines in your spare time in the past year?,” is situated in non-physical spaces which characterized by autonomy, flexibility, social interaction, and contextual relevance. Thus, we think this question refer to the informal learning among older adults.

Based on the research objectives of this study, the dependent variable is classified into two categories: One is a binary discrete variable, namely “whether they participate in informal learning?” which primarily examines the willingness of older adults to participate in informal learning. If an older adult answered “yes,” we assigned a value of “1.” Otherwise, we assigned a value of “0.”

The other one is an ordered discrete variable, namely “the frequency of participation in informal learning,” which primarily examines the frequency of informal learning participation among older adults. If an older adult answered “never,” we assigned a value of “1”; if the old adults answered “rarely,” we assigned a value of “2”; if the old adults answered “sometimes,” we assigned a value of “3”; if the old adults answered “often,” we assigned a value of “4”; if the old adults answered “very often,” we assigned a value of “5.”

### 5.3 Limitations of the dependent variable measurement

We explicitly characterize our measurement as a proxy indicator with three specific limitations:

Limitation 1: Construct underrepresentation. The single-item reading measure captures only literacy-based, individual, indoor informal learning. It does not assess:

- Digital informal learning (e.g., smart-phone-based health information seeking, WeChat group learning).
- Social informal learning (e.g., knowledge exchange in social gatherings, family storytelling).
- Experiential informal learning (e.g., learning through travel, volunteering, craft practice).

Implication: Our 18.47% participation rate should be interpreted as a lower-bound estimate of literacy-based informal learning, not the full scope of older adults’ informal learning engagement.

Limitation 2: Conflation of learning with entertainment. The survey item does not distinguish:

- Learning-oriented reading: Current affairs, health information, practical skills (e.g., cooking, gardening guides).
- Entertainment-oriented reading: Novels, entertainment news, leisure magazines.

Mitigation: We partially address this by noting that even entertainment reading may contribute to cognitive engagement and cultural participation, which are valued outcomes in gerontological education (Biesta, 2011b). However, we cannot isolate “pure learning” from “leisure consumption.”

Limitation 3: Literacy bias and digital exclusion. The measure systematically excludes:

- Illiterate or low-literacy older adults (27.54% of our sample reported no formal education).
- Non-readers who learn through oral/aural channels (radio, TV, conversation).
- Digital learners whose primary informal learning occurs via mobile devices.

Sensitivity check: We re-estimated all models excluding the illiterate group (*n* = 1,291). Results remained substantively unchanged (see Supplementary Appendix C), suggesting that within the literate population, the measure captures meaningful variation. However, this does not resolve exclusion of non-reading learning forms among the literate.

### 5.4 Independent variables

According to Bandura’s (1986) Triadic Reciprocal Determinism of Social Cognitive Theory, this study considers that informal learning of older adults is influenced by the interaction and mutual effects of personal factors, environmental factors, and behavioral factors.

In terms of individual factors, the variables mainly include age, gender, education level, marital status, rural/urban status, and health status of older adults; different individuals have different perceptions of participation in learning.

In terms of environmental factors, the variables mainly include economic income, social interactions, and attitudes towards society.

In terms of behavioral factors, the variables mainly include the mastery of skills by older adults (such as their Mandarin proficiency), psychological state, and sense of wellbeing.

See Table 2 for specific assignments.

**TABLE 2:** Description statistics of main variables.

*N* = 4688		Variables	Assignment	Frequency	Percentage (%)
Dependent variable	Binary variable	Participate	1	866	18.47
		Non-participate	0	3,822	81.53
	Ordered variable	Never	1	2,934	62.59
		Rarely	2	888	18.94
		Sometimes	3	470	10.03
		Often	4	295	6.29
		Very often	5	101	2.15
Individual factors	Gender	Male	1	2,207	47.09
		Female	0	2,481	52.91
	Age	Younger age group: 60–70 (including 60)	1	2,657	56.68
		Middle age group: 70–80 (including 70)	2	1,410	30.08
		Older age group: 80-(including 80)	3	621	13.25
	Urban/rural status	Rural	0	3,569	76.14
		Urban	1	1,119	23.86
	Education level	None	1	1,291	27.54
		Primary school	2	1,372	29.27
		Junior high school	3	1,028	21.93
		High school/vocational secondary school	4	688	14.68
		College and above	5	309	6.59
	Marital status	Without a spouse	0	1,301	27.74
		With a spouse	1	3,387	72.26
	Self-rated health status	Very unhealthy	1	290	6.19
		Relatively unhealthy	2	1,175	25.06
		Average	3	1,343	28.64
		Relatively healthy	4	1,511	32.24
		Very healthy	5	369	7.86
Environmental factors	Income level	Far below the average of their residential area	1	480	10.24
		Below average of their residential area	2	1,699	36.25
		At average level of their residential area	3	2,189	46.7
		Above average of their residential area	4	305	6.51
		Far above average of their residential area	5	14	0.3
	Trust in others	Mostly untrustworthy	1	140	2.98
		Many untrustworthy	2	603	12.85
		Half trustworthy and half untrustworthy	3	569	12.15
		Many trustworthy	4	2,665	56.86
		Mostly trustworthy	5	711	15.17
	The frequency of social interaction	Never	1	703	15
		Rarely	2	1,551	33.08
		Sometimes	3	1,154	24.61
		Often	4	961	20.5
		Very often	5	319	6.81
Cognitive factors	Subjective wellbeing	Very unhappy	1	63	1.34
		Relatively unhappy	2	287	6.13
		Neither happy nor unhappy	3	543	11.59
		Relatively happy	4	2,722	58.05
		Very happy	5	1,073	22.88
	Mandarin proficiency	Cannot understand at all	1	171	3.65
		Poor	2	779	16.62
		Average	3	1,411	30.1
		Good	4	1,476	31.49
		Very good	5	851	18.14
	Depression status	Always	1	80	1.71
		Often	2	403	8.59
		Sometimes	3	1,118	23.84
		Rarely	4	1,752	37.38
		Never	5	1,335	28.47

## 6 Results

The following section includes two subsections, each addressing one of the research questions. The first section is the Cross-tabulation results. It describes older adults’ current status and willingness in informal learning.

The second section is the Multiple Regression Analysis results. It describes the influencing factors and the differences of the willingness and enthusiasm among older learners’ Individual Factors (IF), Environmental Factors (EF), and Cognitive Factors (CF). And then we can answer the second research question.

### 6.1 Cross-tabulation results

**Finding 1: Prevalence of Literacy-Based Informal Learning.**

Among 4,688 older adults, 866 (18.47%) reported any reading activity in the past year. This represents manifest participation in literacy-based informal learning—not total informal learning engagement, which would likely be higher if digital, social, and experiential forms were captured.

Among participants, 470(54.28%) engaged in low-frequency participation (rarely or sometimes), while 396(45.72%) reported high-frequency participation (often or very often). This distribution suggests that even among those who read, intensity is modest, with fewer than 3% of the total sample (101 individuals) reporting very frequent engagement.

**Finding 2: After conducting cross-tabulation analysis between the dependent variables and demographic factor variables, variables such as gender, urban/rural status, marital status, education level, and health status were found significantly influence the willingness of older adults individuals to participate.**

Regarding gender, men demonstrate a higher willingness to participate than women. Concerning urban/rural status, urban older adults exhibit a stronger willingness to participate than rural ones. Regarding marital status, older adults with spouses demonstrate a higher willingness to participate compared to those without spouses. Regarding education level, older adults with a high school education or above are more willing to participate in informal learning. As for physical health status, the willingness to participate shows an inverted “U-shaped” curve. Older adults in the best health condition show the least willingness to participate.

**Finding 3: Age-Group Variation in Participation Enthusiasm.**

Participation frequency varies significantly across age groups (χ^2^ = 22.31, *p* < 0.05, Table 3). The 60-70 age group reports the highest rates of moderate-frequency participation (“sometimes”/”often”), while the 80+ group concentrates in the “never” category.

**TABLE 3A:** Cross-tabulation analysis of participation willingness (binary variable).

Independent variable	Category	Participate *N* (%)	Non-participate *N* (%)	χ ^2^
Gender	Male	452 (20.5)	1,755 (79.5)	15.23***
	Female	414 (16.7)	2,067 (83.3)	
Age group	60–70 years	542 (20.4)	2,115 (79.6)	12.45**
	70–80 years	231 (16.4)	1,179 (83.6)	
	80+ years	93 (15.0)	528 (85.0)	
Household registration	Rural	587 (16.4)	2,982 (83.6)	28.76***
	Urban	279 (24.9)	840 (75.1)	
Marital status	With spouse	712 (21.0)	2,675 (79.0)	32.18***
	Without spouse	154 (11.8)	1,147 (88.2)	
Education level	No formal education	89 (6.9)	1,202 (93.1)	285.40***
	Primary school	186 (13.6)	1,186 (86.4)	
	Junior high school	228 (22.2)	800 (77.8)	
	Senior high/vocational	224 (32.6)	464 (67.4)	
	College and above	139 (45.0)	170 (55.0)	
Health status	Very unhealthy	35 (12.1)	255 (87.9)	18.62***
	Relatively unhealthy	162 (13.8)	1,013 (86.2)	
	Average	237 (17.6)	1,106 (82.4)	
	Relatively healthy	316 (20.9)	1,195 (79.1)	
	Very healthy	116 (31.4)	253 (68.6)	

***P < 0.01, **p < 0.05, *p < 0.10. **χ^2^** indicates the chi-square values for “Participation in informal learning.”

**TABLE 3B:** Cross-tabulation analysis of participation enthusiasm (ordinal variable).

Independent variable	Never	Rarely	Sometimes	Often	Very often
	Participate *N* (%)	Participate *N* (%)	Participate *N* (%)	Participate *N* (%)	Participate *N* (%)
Gender (χ2= 18.42***)					
Male	1,334 (60.4)	455 (20.6)	253 (11.5)	137 (6.2)	28 (1.3)
Female	1,600 (64.5)	433 (17.5)	217 (8.7)	158 (6.4)	73 (2.9)
Age group (χ2 = 22.31***)					
60–70 years	1,560 (58.7)	512 (19.3)	318 (12.0)	194 (7.3)	73 (2.7)
70–80 years	918 (65.1)	253 (17.9)	124 (8.8)	79 (5.6)	36 (2.6)
80+ years	456 (73.4)	123 (19.8)	28 (4.5)	22 (3.5)	8 (1.3)

***P < 0.01, **p < 0.05, *p < 0.10. **χ^2^** indicates the chi-square values for “Frequency of participation in informal learning.”

Interpretive caution: This pattern may partially reflect the larger sample size of the 60–70 age groups (56.68% of total sample). Alternative interpretations—such as cohort-specific educational advantages or life course timing—cannot be empirically tested with these cross-sectional data. We therefore report this as a descriptive association requiring longitudinal verification.

**Conclusion: the willingness of older adult individuals to participate in informal learning is not high, and their enthusiasm for participation is not high.**

### 6.2 Multiple regression analysis

#### 6.2.1 Analysis of the participation willingness in informal learning

The results of the binary logistic regression analysis for the dependent variable “whether they participate in Informal Learning” are presented in Table 4. Three variables are analyzed in three models: Model 1 includes demographic variables, Model 2 includes cognitive variables in Model 1, and Model 3 includes behavioral variables in Model 2.

**TABLE 4:** Binary logistic regression analysis.

Dependent variable: whether to participate in informal learning		Model 1		Model 2		Model 3	
*N* = 4,688		OR	SE	OR	SE	OR	SE
Individual factor	Gender	1.551***	−0.109	1.395**	−0.111	1.387**	−0.111
	Age	1.195**	−0.078	1.329***	−0.08	1.323***	−0.08
	Education level	1.027***	−0.005	1.022**	−0.005	1.022**	−0.005
	Health status	1.033**	−0.016	1.038**	−0.015	1.038**	−0.015
	Urban/rural status	1.002	−0.011	1.001	−0.012	1.001	−0.012
	Marital status	1.853***	−0.142	1.729***	−0.143	1.714***	−0.143
Cognitive factor	Sense of happiness	–	–	1.026**	−0.01	1.029**	−0.011
	Mandarin proficiency	–	–	1.647***	−0.058	1.65***	−0.058
	Psychological depression	–	–	1.005	−0.01	0.999	−0.012
Environmental factor	Economic status	–	–	–	–	1.004	−0.004
	Trust in others	–	–	–	–	0.983	−0.015
	Social interaction	–	–	–	–	1.027**	−0.013
Log likelihood		2575.466		2484.848		2478.609	
R^2^		0.015		0.034		0.035	
AIC		5,164.93		4,985.70		4,975.22	
BIC		5,210.25		5,042.01		5,042.53	

***P < 0.01, **p < 0.05, *p < 0.10.

Finding 1: In Model 1, among factors associated with participation willingness, all variables except urban/rural status demonstrated statistically significant associations.

Specifically, the odds of participation willingness among men were 1.551 times higher than among women, and those with a spouse showed 1.853 times higher odds compared to those without a spouse. Additionally, age, education level, and health status showed positive associations with participation willingness.

Finding 2: the sense of happiness to life and proficiency in Mandarin communication significantly affect the willingness of older adults participating in learning.

After controlling individual variables, Model 2 introduced cognitive factor variables to examine the influence of cognitive factors on the willingness of older adults to participate in learning.

Older adults with stronger sense of happiness to life and better Mandarin communication proficiency are more willing to engage in learning, with their willingness increasing by 1.026 times and 1.647 times, respectively. This indicates that cognitive abilities are important factors determining the spontaneous participation of older adults in informal learning. The variable of psychological depression does not have statistical significance influence on older adults’ willingness to participation in learning. This suggests that the willingness of older adults to participate in learning does not influenced by their emotional state. A reasonable explanation for this phenomenon is that psychological issues can be addressed through various means, although psychological issues vary in severity degree. Some can be resolved through self-regulation, while others may seek for peer support or even consult a psychologist. Therefore, psychological factors are not sufficient and necessary conditions in analyzing factors influencing older adults’ participation willingness. On the contrary, active participation in learning can significantly improve the psychological health of older adults. This highlights the crucial role of actively promoting older adult education to improve the mental health of the aging population.

Finding 3: economic status and trust in others do not significantly affect the participation willingness of older adults.

Model 3, built upon Model 2 and incorporates environmental factor variables to examine the influence of the environment on older adults’ participation willingness in learning.

This conclusion contradicts with general research findings on older adult learning from other scholars. As mentioned before, informal learning has its uniqueness. It is an instinctual behavior. The acquisition of knowledge and how much it acquires does not change with one’s economic status or the degree of trust in others. In Model 3, the frequency of social interaction significantly influences the participation willingness of older adults, with a 1.027 times increase in willingness for every increase in social interaction frequency. That is, the more older adults interact with the society, the more willing they participate in learning. Overall, the participation willingness of older adults is not closely related to the external environment, but is closely associated with interpersonal interactions. This also confirms a characteristic of informal learning: informal learning is a lifelong learning process that requires individuals to continually acquire and accumulate knowledge, skills, attitudes, and viewpoints from daily social interaction and living environment.

#### 6.2.2 Analysis of active participation in informal learning

Finding 1: From the ordinal multinomial logistic regression equation in Table 5, it can be observed that gender, urban/rural status, Mandarin communication ability, and participation in social interaction are all statistically significant at each participation level.

**TABLE 5:** Multinomial ordered logistic regression: determinants of participation enthusiasm.

*N* = 4,688	Rarely	Sometimes	Often	Very often
	OR (S.E.)	OR (S.E.)	OR (S.E.)	OR (S.E.)
Individual factors				
Male (ref: female)	1.651*** (0.081)	1.796*** (0.107)	1.836*** (0.131)	2.092*** (0.232)
Age group (ref: 60–70 years)				
— 70–80 years	0.892 (0.065)	0.845* (0.072)	1.026 (0.089)	1.030** (0.015)
— 80+ years	0.756** (0.078)	0.698*** (0.082)	1.026 (0.102)	1.030** (0.015)
Education level	0.737*** (0.098)	0.765** (0.131)	0.593** (0.169)	0.568 (0.302)
Health status	1.002 (0.009)	1.013 (0.007)	1.024*** (0.007)	1.015 (0.012)
Urban hukou (ref: rural)	1.082** (0.032)	1.098** (0.034)	1.114*** (0.034)	1.079 (0.070)
With spouse (ref: without)	0.997 (0.006)	1.015 (0.007)	1.026** (0.009)	1.030** (0.015)
Cognitive factors				
Life satisfaction	1.021 (0.014)	1.043** (0.015)	1.041** (0.017)	1.061*** (0.015)
Mandarin Proficiency	1.334*** (0.041)	2.186*** (0.049)	2.177*** (0.051)	2.218*** (0.052)
Depression status	0.983 (0.017)	1.000 (0.013)	1.004 (0.013)	0.989 (0.029)
Environmental factors				
Economic status	0.998 (0.004)	1.004 (0.004)	1.003 (0.005)	1.009 (0.008)
Social trust	0.990 (0.007)	0.960 (0.028)	0.978 (0.019)	0.975 (0.029)
Social interaction frequency	1.096** (0.030)	1.099** (0.033)	1.104** (0.034)	1.137*** (0.032)
Model fit				
Log likelihood		−2484.85***		
Nagelkerke R^2^		0.156		
Brant test		χ^2^ = 23.47		

*N* = 4,688; reference category is “never participate”; OR, odds ratio; S.E., robust standard error; ***P < 0.01, **p < 0.05, *p < 0.10. ^1^Data were collected on June, 2018. Source URL: http://cgss.ruc.edu.cn/English/Home.htm

They have a positive impact on the active participation of older adults in informal learning. Specifically, men, urban older adults, and those with strong social interaction skills exhibit higher levels of participation enthusiasm. It should be noted that the Mandarin proficiency has the most significant influence, that is, in each frequency level, stronger Mandarin communication skills correspond to a respective increase in participation enthusiasm which are 1.334, 2.186, 2.177, and 2.218. This is because older adults with weaker Mandarin proficiency tend to have lower literacy rates and weaker information acquisition abilities, leading to the lack of basic communication skills and forming a vicious cycle of “inability to communicate → unwillingness to communicate → poor communication abilities→ more unwillingness to communicate.” This underscores the importance of strong language proficiency, especially a widely spoken language like Mandarin, in enhancing the physical and mental well-being as well as social dignity of older adults.

**Finding 2:** **The effect of marital status on participation enthusiasm varies by frequency level, with significant positive effects emerging at higher participation intensities.**

In the multinomial ordered logistic regression (Table 4), marital status shows no significant association with low-frequency participation (“rarely”: OR = 0.997, *p* = 0.617; “sometimes”: OR = 1.015, *p* = 0.617). However, at higher frequency levels, significant positive effects emerge (“often”: OR = 1.026, *p* = 0.004; “very often”: OR = 1.030, *p* = 0.041). This indicates that having a spouse is associated with greater odds of frequent participation, but not with occasional participation.

Apparent contradiction with binary model and mechanism explanation: This finding appears inconsistent with the binary logistic regression (Table 4), where marital status shows a strong positive effect on participation willingness (OR = 1.714, *p* < 0.001). However, this is not a true contradiction but reflects differentiated mechanisms at the extensive versus intensive margins:

- Extensive margin (participation willingness): Marriage reduces barriers to initial participation through social integration and spousal encouragement, manifesting as a positive effect.
- Intensive margin (participation enthusiasm): Among those already participating, married individuals face domestic time competition (spousal care, household maintenance) that constrains very frequent engagement; conversely, unmarried individuals—particularly the widowed—may use intensive informal learning as compensatory social engagement to address loneliness and fill time voids.

This interpretation is supported by interaction effect analysis (see Supplementary Appendix D): unmarried individuals show a stronger driving effect of social interaction frequency on high-frequency participation (interaction OR = 1.342, *p* < 0.01), indicating their reliance on intensified social networks to compensate for missing spousal support.

**Finding 3: The age variable shows statistically positive significance in the categories of “often participation” and “very often participation,” with an increase of 1.026 times and 1.03 times, respectively.**

However, it does not exhibit statistical significance in the categories of “sometimes participation” and “rare participation.” This indicates that older individuals tend to have a stronger enthusiasm for participation. This aligns with the current situation in formal education of older adults, where the age distribution of students is characterized by a narrow range at both ends and a larger proportion in the middle. Specifically, individuals aged 56–70 accounts for 59.18% of students in the universities of older adults, while the participation rate of the oldest individuals is only 12.95%. Older adults individuals who cannot access to formal learning have no choice but to engage in informal learning based on their daily lives.

**Finding 4: concerning health status, among older adult individuals who self-rate themselves healthy, their participation frequency of “sometimes participation” and “often participation” is high, but the frequency of “very often participation” is not high.**

One possible explanation is that older adult learning is not a conscious, skill-oriented and survival required learning, but rather a casual and practice-oriented learning. This type of learning characterized by “economic benefit”: individuals only engage in learning if they perceive it as valuable, otherwise they do not want to learn. For older adults, the purpose of participation in learning is not to acquire higher survival skills, but primarily for leisure and entertainment. Therefore, good health status can promote participation enthusiasm. However, it is not necessary to rely on health status to increase participation enthusiasm; in other words, they are sufficient conditions but not necessary conditions.

**Finding 5: Interaction between marital status and social interaction frequency explains variation in participation enthusiasm.**

To investigate potential mechanisms underlying the marital status effects, we estimated interaction models (Supplementary Appendix Table D2). Results reveal a significant positive interaction between marital status and social interaction frequency at higher participation levels (“often”: interaction OR = 1.203, *p* < 0.05; “very often”: interaction OR = 1.342, *p* < 0.01).

Stratified analysis (Supplementary Appendix Table D3) shows that:

- Among unmarried older adults, social interaction frequency has a stronger effect on high-frequency participation (“very often”: OR = 1.102, *p* < 0.05).
- Among married older adults, this effect is attenuated (“very often”: OR = 1.186, *p* < 0.001) but the baseline participation level is higher.

Interpretation: This pattern supports a compensatory mechanism hypothesis: unmarried older adults rely more heavily on social interaction to drive their informal learning engagement, using learning as a substitute for spousal social support. Married individuals have higher baseline participation due to spousal encouragement and shared resources, but their participation is less sensitive to external social interaction because their primary social needs are met within marriage.

### 6.3 Caution in interpretation

Given the cross-sectional nature of CGSS data, all reported associations should not be interpreted as causal relationships. The observed relationships may reflect: (a) genuine predictive effects, (b) reverse causality (e.g., active learning improving health or social skills), or (c) unobserved confounding variables. Longitudinal designs are needed to establish causal inference.

## 7 Conclusion

This study examines the participation willingness and participation enthusiasm of older adults in informal learning as well as the influential factors, using data from the Chinese General Social Survey (CGSS) conducted in 2018. The research yields three primary conclusions:

Firstly, older adults demonstrate a relatively low willingness to participate in informal learning, and their enthusiasm to participate in informal learning is not strong (Hachem, 2023b).

Secondly, in terms of individual factors, men and older adults with a spouse demonstrate a strong willingness to participate. Age, education level, and health status positively influence the participation willingness of older adults. From a cognitive perspective, sense of satisfaction towards life and Mandarin proficiency in communication significantly positively affect their participation willingness. From a behavioral standpoint, social interaction significantly promotes the participation willingness of older adults.

Thirdly, in terms of participation enthusiasm, gender, urban/rural status, marital status, Mandarin proficiency, and social interaction exhibit significant positive effects, though these effects vary by participation frequency. Notably, marital status shows differential effects: it strongly predicts participation willingness (binary outcome) and high-frequency enthusiasm (“often”/”very often”), but does not distinguish low-frequency participants from non-participants. This suggests marriage operates as a resource facilitator for sustained engagement rather than a barrier to initial participation. The interaction between marital status and social interaction (Supplementary Appendix D) further indicates that unmarried older adults compensate through intensified social engagement, highlighting the adaptability of informal learning patterns to diverse life circumstances.

## 8 Implications

Caution in interpretation: Given the cross-sectional nature of CGSS data, all reported associations should not be interpreted as causal relationships. The observed relationships may reflect: (a) genuine predictive effects, (b) reverse causality (e.g., active learning improving health or social skills), or (c) unobserved confounding variables. Longitudinal designs are needed to establish causal inference.

### 8.1 Specific theoretical contributions

This study makes distinct contributions to three theoretical domains. However, all contributions should be interpreted with caution due to the proxy indicator limitation (literacy-based informal learning only).

Contribution 1: preliminary test of social cognitive theory in informal learning contexts (subject to further validation).

Our findings suggest that, in the context of literacy-based informal learning, cognitive factors (particularly linguistic capability) exhibit stronger explanatory power than environmental factors (except social interaction). This pattern differs from findings in formal education research, where economic capital and institutional resources are often more critical. However, we emphasize that this conclusion is constrained by our proxy indicator (reading behavior). Digital or social informal learning may exhibit different factor structures. We therefore present this finding as exploratory evidence rather than definitive conclusion, pending validation with more comprehensive measurement instruments.

Contribution 2: Refinement of third age learning theory with contextual caveats

Laslett’s (1989) Third Age theory posits post-retirement as a period of active self-realization through learning. Our findings suggest that this assumption may be contextually contingent: only 18.47% of Chinese older adults in our sample participated in literacy-based informal learning, indicating that structural barriers (educational legacy, linguistic marginalization) may override developmental readiness. However, we acknowledge that this low participation rate may partially reflect measurement artifact—our literacy-based indicator fails to capture oral or digital learning forms. We therefore propose a resource paradox hypothesis as a tentative explanation requiring further testing.

Contribution 3: Integration of human capital and cultural capital theories (tentative).

Contribution 3: Integration of human capital and cultural capital theories.

Our findings integrate insights from economics (Becker, 1964) and sociology (Bourdieu, 1986):

- Economic capital shows null effects: Unlike formal education research where income strongly predicts participation, informal learning shows no significant income gradient. This suggests informal learning operates as “low-threshold human capital accumulation” that is economically accessible but still stratified by cultural capital (education, language, social connections).
- Social capital operates through interaction frequency, not trust: Contrasting with Putnam’s (2000) emphasis on generalized trust, we find that behavioral social engagement (interaction frequency) matters more than attitudinal social capital (trust). This refines understanding of how social networks facilitate learning—through enacted relationships rather than abstract community cohesion.

### 8.2 Practical implications

Current older adult education source is characterized by tight supply and demand, uneven distribution and significant regional disparities. Under this background, positively developing informal education among older adults is an effective measure to alleviate this contradiction.

Firstly, measures to increase participation willingness should target men, middle-to-lower age groups, individuals with spouses (who show higher participation odds), those with some education, and relatively healthy individuals. However, for participation enthusiasm, the target population differs: while married individuals show higher odds of frequent participation, unmarried individuals with strong social interaction networks also achieve high engagement levels. This suggests dual policy tracks:

- For married older adults: leverage spousal support to sustain high-frequency participation.
- For unmarried older adults: strengthen community social infrastructure to provide compensatory engagement opportunities.

Secondly, factors such as strong language skills, a healthy mindset, stable economic security as well as a harmonious, friendly living environment are positive influences older adults’ participation in learning. In practice, we must not only focus on the cognitive literacy of older individuals but also consider the impact of the surrounding environment, particularly their subjective perceptions of wellbeing, trust, and other aspects of the mental environment. The formation of a positive mental state or environment relies on continuous attention to the daily lives of older adults. From this perspective, promoting older adult participation in learning is an ongoing effort that must begin with attention to the finer details of everyday life. Therefore, we should fully utilize the interest-based social interaction which is about health among older adults. This can be achieved through various means such as family, friends, colleagues, neighbors, libraries, television, Internet and other mass media. Seeking to create a learning ecosystem during leisure time and nurturing learning communities among older adults in order to provide more opportunities of informal learning for older adults, thereby enhancing their participation enthusiasm.

Thirdly, we should expand the supply of older adult education as possible as we can. On the one hand, we should expand the formal learning system primarily represented by universities for older adults and community colleges. On the other hand, for the informal learning system, we should rely on various social organizations to attract older adults individuals. Only in this way will older adults have more opportunities to participate in learning.

### 8.3 Implications for conceptualizing informal learning in aging research

Our findings must be interpreted within the constraints of a literacy-based proxy measure. The strong predictive effects of Mandarin proficiency and urban residence likely reflect, in part, measurement artifact—our indicator is more sensitive to the learning forms accessible to linguistically-capable, urban-dwelling older adults.

Had we measured informal learning more comprehensively (including digital, social, and experiential forms), we might observe:

- Smaller urban-rural gaps: Rural older adults may engage in rich oral/aural and community-based learning invisible to our measure.
- Different educational gradients: Digital learning might show flatter socioeconomic gradients due to mobile device penetration.
- Stronger social interaction effects: Social learning might emerge as a distinct dimension, not merely a predictor of reading-based learning.

Recommendation for future research: Develop and validate multi-dimensional informal learning scales for Chinese older adults that capture:

- Literacy-based learning (reading, writing).
- Digital learning (online information seeking, mobile courses).
- Social learning (family knowledge exchange, peer learning).
- Experiential learning (practice-based skill development).
- Community-based learning (organized non-curricular activities).

## 9 Limitations and future research

### 9.1 Measurement limitations: the proxy indicator problem

The most significant limitation of this study is the narrow operationalization of informal learning. We have attempted to mitigate this through:

- Transparent disclosure (this section, and repeated caveats throughout).
- Theoretical boundary specification (framing findings as “literacy-based informal learning”).
- Sensitivity analysis (excluding illiterate group; see Supplementary Appendix C).
- Interpretive caution (avoiding claims about “all informal learning”).

However, these mitigations do not eliminate the fundamental constraint. Our findings pertain to a specific modality of informal learning that may be increasingly anachronistic as digital platforms transform older adults’ learning ecosystems. The 2018 data collection predates the COVID-19 pandemic, which accelerated digital adoption among older adults, and the explosive growth of short-video platforms (e.g., Douyin/Kuaishou) as informal learning channels.

Readers should generalize our findings with caution to:

- Contemporary contexts where digital informal learning is prevalent.
- Populations with strong oral/aural learning traditions (e.g., ethnic minority communities).
- Future cohorts of older adults with higher digital literacy.

### 9.2 Omitted variable: digital access and literacy

A significant limitation of our model is the absence of digital technology variables. The 2018 CGSS wave did not include comprehensive measures of internet access, smart phone use, or digital skills—factors increasingly central to informal learning ecosystems (Lü et al., 2023). This omission is particularly consequential given China’s rapid digitalization and the COVID-19 pandemic’s subsequent transformation of learning modalities.

Impact on findings:

- Our strong Mandarin proficiency effect may partially proxy for digital literacy, as Mandarin competence correlates with formal education, which predicts digital skill acquisition.
- The urban-rural participation gap may be underestimated if digital access barriers are unobserved—rural areas face systematic digital infrastructure deficits.
- We cannot assess whether digital informal learning (e.g., health information seeking via WeChat, short-video learning) substitutes for or complements the reading-based learning we measure.

Implications for interpretation: Our findings pertain to pre-digital or para-digital informal learning and may not generalize to contemporary learning environments where digital platforms dominate. Future research must explicitly integrate digital access, skills, and usage patterns.

### 9.3 Future research

This study utilized multivariate statistical methods to explore the willingness and participation enthusiasm of older adults in informal learning, drawing preliminary conclusions with universal implications. But this study has some limitations, and the findings of this study should be interpreted in light of these.

First, the analysis based on the 2018 CGSS data reflects certain aspects of older adults’ participating in informal learning. However, in recent years, the rapid development of the Internet and other technologies has gradually permeated the lives of older adults. The progress in technology has brought about continuous updates in the content and forms of learning, which can be described as ever-changing. Therefore, exploration based on more up-to-date data will be a direction for future research.

Second, this study employs cross-sectional data, which demonstrates the correlation between older adults’ participation outcomes in informal learning with both cognitive and environmental factors. However, if the panel data were used, it would be possible to more deeply reveal causal inferences.

Third, in the study, we only examined the direct effects of three factors (Individual Factors, Environmental Factors and Cognitive Factors) on participation willingness and participation enthusiasm. Are there any indirect effects of these factors on participation willingness and participation enthusiasm? If there are indirect effects, how and to what extend these factors influence participation willingness and participation enthusiasm? In summary, there are still many unresolved issues in research on older adult participation in informal learning, and these need to be further explored in future studies.

## Data Availability

The original contributions presented in this study are included in the article/Supplementary material, further inquiries can be directed to the corresponding authors.
