# Supplementary material for: An empirical study of older adults’ willingness and enthusiasm for informal learning
Source: Front Psychol. 2026 Aug 6;17:1782394. doi: 10.3389/fpsyg.2026.1782394 (PMC13492197; doi:10.3389/fpsyg.2026.1782394)
Supplement: Supplementary file 1 [file Supplementary_file_1.doc]

# Appendix

## Appendix A Summary of Regression Model Diagnostic Statistics

Table A1 Summary of Regression Model Diagnostic Statistics

| **Diagnostic Item** | **Test/Index** | **Result** | **Conclusion** |
| --- | --- | --- | --- |
| Multicollinearity | VIF Range | 1.02–2.34 | No severe multicollinearity |
| Proportional Odds Assumption | Brant test (overall) | χ²=23.47, p=0.214 | Assumption holds |
| Binary Model Fit | Hosmer-Lemeshow test | p > 0.05 | Good model calibration |
| Ordinal Model Fit | Likelihood ratio test | p < 0.001 | Model significant overall |
| Model Comparison | AIC/BIC | Reported for each model | Model 3 is optimal |

*Note. VIF = Variance Inflation Factor.*

## Appendix B Replication Guide

B.1 Data Access and Documentation

Primary Data Source: China General Social Survey (CGSS) 2018

| **Item** | **Details** |
| --- | --- |
| Data source | Chinese General Social Survey (CGSS), 2018 wave |
| Data provider | National Survey Research Center, Renmin University of China |
| Official website | <http://cgss.ruc.edu.cn/English/Home.htm> |
| Data collection period | June–September 2018 |
| Sampling frame | 2009 National Population Census data |
| Sample size (total) | 12,000 households, 11,847 individual respondents |
| Our analytical sample | 4,688 respondents aged 60+ years |

Data Access Procedure:

Register an account at CGSS official website

Submit research proposal and data use agreement

Download SPSS format datasets after approval (typically 3–5 working days)

Cite data source: "This research uses data from the Chinese General Social Survey (CGSS)."

B.2 Sample Selection Criteria and Code

Inclusion Criteria:

Age: 60 years or older at time of survey

Valid response on dependent variable (informal learning participation)

Complete data on key independent variables (gender, education, health, Mandarin proficiency)

Exclusion Criteria:

Missing values on dependent variable (n = 234)

Missing values on >50% of key independent variables (n = 127)

B.3 Variable Coding Manual

| **Variable Name** | **Original CGSS Item** | **Coding Scheme** | **Values** | **Missing Treatment** |
| --- | --- | --- | --- | --- |
| **Dependent Variables** | | | | |
| Informal  learning | a36 | Binary | 0=Never, 1=Any participation | Listwise deletion |
| Informal  freq | a36 | Ordinal | 1=Never, 2=Rarely, 3=Sometimes, 4=Often, 5=Very often | Listwise deletion |
| **Individual Factors** | | | | |
| Male | a2 | Binary | 0=Female, 1=Male | Listwise deletion |
| Age group | age | Categorical | 1=60-69, 2=70-79, 3=80+ | Calculated from age |
| Education | a7a | Ordinal | 1=None, 2=Primary, 3=Junior high, 4=Senior high/vocational, 5=College+ | Listwise deletion |
| Health | a15 | Ordinal | 1=Very unhealthy to 5=Very healthy | Listwise deletion |
| Urban | samp_type | Binary | 0=Rural, 1=Urban | Listwise deletion |
| Married | a69 | Binary | 0=No spouse, 1=With spouse | Listwise deletion |
| **Cognitive Factors** | | | | |
| Life satisfaction | a36a | Ordinal | 1=Very unhappy to 5=Very happy | Mean imputation if missing<5% |
| Mandarin | a46 | Ordinal | 1=Cannot understand to 5=Very good | Listwise deletion |
| Depression | a17 | Ordinal (reversed) | 1=Always depressed to 5=Never | Mean imputation if missing<5% |
| **Environmental Factors** | | | | |
| Income | a8a | Ordinal | 1=Far below average to 5=Far above | "Don't know" coded as missing |
| Social trust | a33 | Ordinal | 1=Mostly untrustworthy to 5=Mostly trustworthy | Mean imputation if missing<5% |
| Social interaction | a31 | Ordinal | 1=Never to 5=Very often | Mean imputation if missing<5% |

## Appendix C Sensitivity Analysis

Table C1 Sensitivity Analysis: Excluding Illiterate Group

| **Model** | **Sample** | **N** | **Mandarin Proficiency OR** | **Interpretation** |
| --- | --- | --- | --- | --- |
| Full model | All respondents | 4,688 | 1.650*** | Main finding |
| Excluding illiterate | Education ≥ Primary | 3,397 | 1.523*** | Effect persists, slightly attenuated |
| Excluding primary only | Education ≥ Junior high | 2,025 | 1.380*** | Further attenuation suggests education-Mandarin correlation |
| Urban only | Urban residents | 1,119 | 1.280* | Effect persists but weaker in urban areas |
| Rural only | Rural residents | 3,569 | 1.780*** | Effect stronger in rural areas |

*Note. *** p<0.01, ** p<0.05, * p<0.10.Conclusion: The core finding that Mandarin proficiency predicts informal learning participation is robust across sample restrictions.*

## Appendix D Robustness Checks and Interaction Effects for Marital Status

D.1 Alternative Model Specifications

Table D1 Alternative Specifications: Marital Status Effects on Participation Enthusiasm

| **Model Specification** | **Sample** | **Marital Status OR (Rarely)** | **Marital Status OR (Sometimes)** | **Marital Status OR (Often)** | **Marital Status OR (Very Often)** |
| --- | --- | --- | --- | --- | --- |
| Baseline ordered logit | Full sample (N=4,688) | 0.997 | 1.015 | 1.026** | 1.030** |
| Zero-inflated ordered logit | Full sample | 0.892 | 0.945 | 0.978 | 1.012 |
| Generalized ordered logit | Full sample | 0.987 | 1.023 | 1.031* | 1.038** |
| Multinomial logit (no ordering) | Full sample | 0.954 | 1.008 | 1.042* | 1.051** |
| Excluding widowed only | Married/Divorced/Unmarried (N=3,456) | 1.012 | 1.028 | 1.035** | 1.042** |
| Gender-stratified: Male | Male only (N=2,207) | 1.023 | 1.038 | 1.045* | 1.052** |
| Gender-stratified: Female | Female only (N=2,481) | 0.978 | 0.998 | 1.012 | 1.018 |

*Note. All models control for full set of covariates (IF, CF, EF). *** p < 0.01, ** p < 0.05, * p < 0.10*

D.2 Interaction Effects

Table D2 Interaction Effects: Marital Status × Social Interaction Frequency

| **Participation Level** | **Main Effect: Marital Status** | **Main Effect: Social Interaction** | **Interaction Effect** | **Model Fit (ΔAIC)** |
| --- | --- | --- | --- | --- |
| Rarely vs. Never | 0.892 (0.156) | 1.078* (0.042) | 1.134 (0.089) | -2.3 |
| Sometimes vs. Never | 0.945 (0.142) | 1.082* (0.041) | 1.156* (0.078) | -4.7* |
| Often vs. Never | 0.978 (0.138) | 1.089** (0.040) | 1.203** (0.082) | -8.2** |
| Very Often vs. Never | 1.012 (0.145) | 1.118** (0.039) | 1.342** (0.095) | -12.6*** |

*Note. ORs reported with robust SEs in parentheses. Interaction terms show multiplicative effects. Negative ΔAIC indicates improved model fit with interaction. * p<0.10, ** p<0.05, *** p<0.01.*

D.3 Stratified Analysis by Marital Status

Table D3 Social Interaction Effects Stratified by Marital Status

| **Participation Level** | **Unmarried: Social Interaction OR** | **Married: Social Interaction OR** | **Difference (z-test)** |
| --- | --- | --- | --- |
| Rarely vs. Never | 1.072 (0.048) | 1.089 (0.051) | z=0.24, p=0.81 |
| Sometimes vs. Never | 1.078* (0.045) | 1.098* (0.047) | z=0.31, p=0.76 |
| Often vs. Never | 1.085* (0.044) | 1.128** (0.046) | z=0.72, p=0.47 |
| Very Often vs. Never | 1.102** (0.042) | 1.186*** (0.044) | z=1.45, p=0.15 |

*Note. Models estimated separately for unmarried (n=1,301) and married (n=3,387) subgroups. All models control for IF, CF, EF covariates.*

D.4 Summary

The apparent "contradiction" between binary and ordinal models is resolved through:

- Threshold clarification: Marriage predicts crossing the participation threshold (binary) and sustained high-frequency engagement (ordinal upper categories), but not low-frequency occasional participation
- Mechanism elucidation: Interaction analysis reveals marriage and social interaction as substitute resources for informal learning engagement, with unmarried individuals compensating through intensified social networks
- Robustness verification: Effects persist across model specifications, sample definitions, and stratification approaches
